# Infant gut microbiota coverage by different culture media

**DOI:** 10.1186/s12866-026-04854-7

**Published:** 2026-03-09

**Authors:** Gaurab Aditya Dhar, Heleen Koster, Nienke van Beek, Katri Korpela

**Affiliations:** 1 Human Microbiome Research Program, Research Programs Unit, Faculty of Medicine, University of Helsinki, Helsinki, 00014 Finland; 2 Department of Bacteriology and Immunology, Faculty of Medicine, University of Helsinki, Helsinki, 00014 Finland

**Keywords:** Infant gut microbiota, Culture media, Culturomics, Anaerobic bacterial culture

## Abstract

**Background:**

Culture-independent methods, such as sequencing, have been a breakthrough, but do not allow for complete genetic, biochemical, and phenotypic characterization at strain level. To fully characterize the gut microbiota, effective culture-based methods are needed to complement culture-independent methods.

**Aim:**

This study aims to identify the culture media and supplements to culture a wide range of bacteria from the infant microbiota.

**Methods:**

Faecal samples from a one-year-old Caesarean section born baby, and a two-year-old vaginally born baby were grown in 40 different combinations of growth media, supplements and atmosphere. Colonies taxonomically annotated based on 16S rRNA gene sequences, and a generalized linear model was used to determine the effect of the growth conditions on colony number and species diversity per family.

**Results:**

Rich non-selective media did not support a broad diversity. A combination of selective media preparations targeting different bacterial groups resulted in better coverage of the total community. A combination of 3 formulations was able to capture 80% of the core infant gut genera: modified De Man-Rogosa-Sharpe (mMRS) with mupirocin, Gifu Anaerobic Medium (GAM) with ciprofloxacin and erythromycin and acidified MRS (MRS5.4) in microaerobic conditions. For species-level diversity, MRS5.4 produced the highest diversity of *Bifidobacteriaceae* and *Enterococcaceae*, but other base media did not significantly differ in terms of obtained diversity. However, the addition of specific antibiotics was critical in increasing the observed species-level diversity of bacterial families. Within species, some strains show media preference, and often a combination of media was required to capture the full spectrum of strains.

**Conclusion:**

Using a range of base media, supplements, and growth conditions, we were able to culture a large fraction of the core infant gut bacteria. We find that media specificity of infant gut bacterial isolates often deviates from existing information, and our findings could aid choice of media and growth conditions in future culture-based studies of the infant gut microbiota.

**Supplementary Information:**

The online version contains supplementary material available at 10.1186/s12866-026-04854-7.

## Background

Trillions of microbes harboured by the human body, influence the homeostatic functioning and overall physiology of the host [1]. These microbial communities, collectively termed as the human microbiota, are located at different regions of the human body but have the highest density inside the gastrointestinal tract, giving rise to the gut microbiota [2]. Comprising of indigenous (or, autochthonous) and transient (or, allochthonous) microbes [3], the microbiota harboured inside the enteric environment forms a complex and dynamic community. Shifts in microbiota composition begin from its inception during host infancy, from exposure to maternally and environmentally sourced microbes, till about two years of age [4]. At this age the infant gut microbiome attains a certain degree of compositional stability [4], although the microbiota continues to evolve throughout childhood and through adolescence towards adulthood [5]. These compositional variations in the infant gut microbiota have been attributed to several host-intrinsic and extrinsic factors such as[6–8], as well as to the maturation of the gut and the immune system. This period of compositional variability in early life microbiome development is of considerable interest as the extensive host-microbiome dialogue during this developmental window influences health outcomes. This plays a critical role in the determination of host health by exercising regulatory function over metabolism, physiology, development and immune function [4, 9–11].

To investigate this crucial host-microbiota dialogue at this developmental juncture, efforts have been directed towards elucidation of the infant gut microbial composition, with perhaps the biggest boost being provided by the advancements in high-throughput Next Generation Sequencing technologies [12]. Indeed, culture-independent techniques have made a significant contribution towards bridging the “Great Plate Count Anomaly”, which is the dichotomy between the number of microbes observed under a microscope and the ones cultured in the laboratory [13], thus boosting the identification of several novel microbes inhabiting the human gut. However, these techniques suffer from certain biases pertaining to differences in DNA extraction and bioinformatic analyses protocols across laboratories [14, 15], as well as the inability to discriminate between live and dead bacteria [16, 17]. In addition, the short read lengths [18] and lack of information on the microbial dark matter [19] pose a challenge in achieving precise taxonomic assignment. Moreover, all the sequencing methods suffer from a depth bias which translates to difficulty in detection of microbes having very low abundances [20]. All these drawbacks have limited its usefulness in enquiring the host-microbe crosstalk and microbial ecology in the gut and have contributed to the renewed interest in culture-dependent techniques [21, 22]. The lack of parity in advancement between culture-based methods and culture-independent workflows has created a wide gap between the isolated and culturable fraction and the detected-yet-unculturable fraction of the gut microbiome [21]. Reports indicate only a 56% commonality between gut microbial members detected through NGS techniques and the known culturable members [23, 24], although with the advent of culturomics, this gap is on a path of gradual diminution [25–27].

Culture-based techniques have two advantages over NGS-based methods. Firstly, culturing aids in the capture of the live component of the microbiota [28, 29] which allow for their preservation as well as the complete genetic, biochemical and phenotypic characterization of these isolates [30, 31], thus facilitating the deciphering of host-microbe and microbial crosstalk networks [32]. Secondly, these methods are necessary in unearthing the gut microbial ecology and its functional implications at the strain-level [33]. Accurate construction of genomes of closely related strains from short-read metagenomic data is not possible due to the similarity of the genomes [34]. For this reason, the sub-species-level diversity within a person has remained elusive. This has become necessary as evidence supporting the importance of strain-level functional diversity within the gut microbiota accumulates. Interaction with the host immune system [35] and ability to modify activity of pharmacological agents [36] have already been shown to vary between strains of the same species. In addition, culture-based exploration of strain-level diversity would allow the estimation of the extent to which sequencing-detected genetic diversity translates into functional differences in different conditions. Support for this approach has been given by the elucidation of differentially occurring strains of the human gut resident *Prevotella copri* based on variation in the eating habits of the host [37, 38]. This study revealed that even though specific polysaccharide utilisation genes were the basis of functional variation in these strains, it was only with isolation of strains followed by in vitro tests that confirmed the ability of these strains to utilise various glycans [39].

To obtain the complete microbial community has been the ultimate goal of culture-based studies and to this end, several studies have contributed to the development of high-throughput culturing methods, ranging from modifications to existing techniques [40, 41] and media [27, 42, 43] to development of new culture conditions [23, 25, 26] and in vitro systems, reviewed in Vrancken et al. [44]. A survey indicated redundancy of using different culture media for gut microbial isolation suggesting the use of a single, defined medium [45], but there have been reports on the utility of different growth media with various modifications for targeted culturing of microbes [27, 42, 46]. This strategy allowed the growth of microbes that generally resist growing in a single medium due to being outcompeted by numerically dominant microbes sharing the same nutritional requirement for growth. Even though studies on the high-throughput microbial culture coupled with identification using genomic methods have been done on the adult gut microbiome, similar studies with respect to the infant gut microbiome have been scarce till date. In this work, we aim to estimate the coverage of different culture media over the infant gut microbiome to identify the most suitable combination of media for infant gut culturomics studies.

## Methods

### Study design

Faecal samples were collected from a Caesarean-born 1-year-old infant and a vaginally born 2-year-old infant and immediately frozen at −20℃. Children at this age harbour both infant-type and adult-type microbes [4], making the results likely applicable to samples from both younger and older children. Buffered peptone water (ISO, Dehydrated, Oxoid™, ThermoFisher Scientific, CM1049B) was prepared according to the manufacturer’s instructions with the prepared peptone water having a pH of 7.0. Samples (10 mg) were homogenized in buffered peptone water (10 mg/ml concentration of faecal material to peptone water) and incubated overnight before plating on agar medium having a specific media formulation and growth condition, summarized in Table 1. An aliquot of the homogenized samples (100 µL) was subjected to ethanol (Berner Medical, 13210124) treatment for one hour, prior to plating to enhance the recovery of spore-forming bacteria. After an incubation of 48 hours, the obtained bacterial colonies were picked individually into 600 µL of Brain-Heart Infusion (BHI) broth in a 96-deep well plate, grown approximately 18 hours at 37°C anaerobically without shaking and subjected to 16S rRNA gene sequence PCR (see Sect. 2.3 below), and 16S rRNA gene sequence Sanger sequencing. The colonies were chosen based on their morphological differences to maximise the diversity of isolation. The number of colonies sequenced per media formulation is shown in Table 1.

**Table 1:** The growth conditions utilized in this work and the number of colonies subjected to 16S rRNA gene sequence Sanger sequencing. For media codes having suffix –candle, the microaerobic growth condition was achieved by using a candle in an air-tight container while for media codes having suffix –anopack, the same was achieved using an anaerobic pack (Oxoid™ AnaeroGen™ 2.5 L Sachet, ThermoFisher Scientific, AN0025A)

Media Code	Growth Media	Added component	Growth condition	No. of colonies sequenced
BBEgent	Bacteroides Bile Esculin Agar	Gentamycin	Anaerobic	29
BHI	Brain-Heart Infusion	-	Anaerobic	28
BHIaerobic	Brain-Heart Infusion	-	Aerobic	14
BHIcandle	Brain-Heart Infusion	-	Microaerobic	4
BHIcipro	Brain-Heart Infusion	Ciprofloxacin	Anaerobic	54
BHIciproeryt	Brain-Heart Infusion	Ciprofloxacin + Erythromycin	Anaerobic	40
BHIethanol	Brain-Heart Infusion	Ethanol pre-treatment	Anaerobic	23
BHIGN	Brain-Heart Infusion	G.N. Anaerobic Supplement	Anaerobic	23
BHIkanvanc	Brain-Heart Infusion	Kanamycin + Vancomycin	Anaerobic	22
BHImucin	Brain-Heart Infusion	Mucin (1%)	Anaerobic	5
GAM	Gifu Anaerobic Medium	-	Anaerobic	31
GAMcipro	Gifu Anaerobic Medium	Ciprofloxacin	Anaerobic	75
GAMciproeryt	Gifu Anaerobic Medium	Ciprofloxacin + Erythromycin	Anaerobic	30
GAMethanol	Gifu Anaerobic Medium	Ethanol pre-treatment	Anaerobic	15
GAMGN	Gifu Anaerobic Medium	G.N. Anaerobic Supplement	Anaerobic	17
GAMmucin	Gifu Anaerobic Medium	Mucin (1%)	Anaerobic	8
MMHMO	Minimal Media	Human Milk Oligosaccharides	Anaerobic	4
Rogosa	De Man-Rogosa-Sharpe	-	Aerobic	35
Rogosaanopack	De Man-Rogosa-Sharpe	-	Microaerobic	13
Rogosacandle	De Man-Rogosa-Sharpe	-	Microaerobic	49
RogosaMet	De Man-Rogosa-Sharpe	Metronidazole	Anaerobic	11
RogosaMetCandle	De Man-Rogosa-Sharpe	Metronidazole	Microaerobic	12
MRSHMO	De Man-Rogosa-Sharpe	Human Milk Oligosaccharides	Anaerobic	16
MRS5.4	De Man-Rogosa-Sharpe (acidified, pH 5.4)	-	Aerobic	52
MRS5.4anopack	De Man-Rogosa-Sharpe (acidified, pH 5.4)	-	Microaerobic	15
MRS5.4candle	De Man-Rogosa-Sharpe (acidified, pH 5.4)	-	Microaerobic	58
MRS5.4cys	De Man-Rogosa-Sharpe (acidified, pH 5.4)	Cysteine hydrochloride	Anaerobic	23
MRS5.4Met	De Man-Rogosa-Sharpe (acidified, pH 5.4)	Metronidazole	Anaerobic	16
MRS5.4MetCandle	De Man-Rogosa-Sharpe (acidified, pH 5.4)	Metronidazole	Microaerobic	38
MRS5.4VanCandle	De Man-Rogosa-Sharpe (acidified, pH 5.4)	Vancomycin	Microaerobic	13
MRS5.4MetVanCandle	De Man-Rogosa-Sharpe (acidified, pH 5.4)	Metronidazole + Vancomycin	Microaerobic	14
h-MRS5.4HMO	De Man-Rogosa-Sharpe (acidified, pH 5.4)	Human Milk Oligosaccharides	Anaerobic	6
mMRSmup	De Man-Rogosa-Sharpe (modified)	Lithium Mupirocin	Anaerobic	57
h-mMRSmupHMO	De Man-Rogosa-Sharpe (modified)	Cysteine hydrochloride + Lithium mupirocin + Human Milk Oligosaccharides	Anaerobic	18
TOS	Transgalactosylated oligosaccharides propionate agar	Lithium Mupirocin	Anaerobic	36
VeilVan	Veillonella Agar Base	Vancomycin	Anaerobic	16
WCA	Wilkins – Chalgren Anaerobic Agar	-	Anaerobic	7
WCAciproeryt	Wilkins – Chalgren Anaerobic Agar	Ciprofloxacin + Erythromycin	Anaerobic	6
WCAGN	Wilkins – Chalgren Anaerobic Agar	G.N. Anaerobic Supplement	Anaerobic	18
WCAmucin	Wilkins – Chalgren Anaerobic Agar	Mucin (1%)	Anaerobic	6

### Selected media and media preparation

The choice of base media was done based on pre-existing studies that identified these media as suitable for isolation and growth of gut microbes [45, 47–50]. Brain Heart Infusion (BHI) media is a nutrient-rich medium that supports the growth of various microorganisms, including those found in the infant gut microbiome. It is especially useful in cultivating fastidious organisms, which require specific nutritional and environmental conditions to thrive [51]. BHI contains infusions from calf brains and beef heart, peptone, dextrose, sodium chloride, and disodium phosphate, providing a comprehensive nutrient profile that promotes microbial growth under both aerobic and anaerobic conditions [52]. Gifu Anaerobic Medium (GAM) is a specialized culture medium used to grow anaerobic bacteria. GAM is particularly effective in cultivating a broad range of anaerobic bacteria from the human gut [53]. A study published in 2020 demonstrated that GAM could successfully culture 72% of the top 56 species listed in the human gut microbial gene catalogue [54]. De Man–Rogosa–Sharpe (MRS) agar is a widely used culture medium for the cultivation of lactic acid bacteria, particularly those from the *Lactobacillaceae* family and the genus *Bifidobacterium*, which are important members of the infant gut microbiome. Wilkins–Chalgren Anaerobic (WCA) Agar is an essential media in clinical research, particularly for isolation and susceptibility testing of anaerobic bacteria [55–57]. Over the years, this medium has sustained its importance as it can grow commonly isolated anaerobes without requiring supplementation of blood and provided reproducible values of MICs for control strains used for testing [55]. Vancomycin agar base (VAB) is used to selectively isolate *Veillonella* with sodium lactate serving as a unique nutritional source and vancomycin as an inhibitory agent [58–60].

To improve the selectivity, modified versions of the base media have been developed to improve the isolation of specific bacteria. For example, adding mupirocin selectively inhibits non-bifidobacterial strains, enhancing the recovery of *Bifidobacterium *[61]. A study by Novakova et al. [62] utilised WCA supplemented by mupirocin and 8-hydroxyquinoline as an anti-clostridial agent to enhance recovery of bifidobacteria from faecal samples of infants born by Caesarean section as these samples are generally bifidobacteria-deficient. By adding Human Milk Oligosaccharides (HMO) to the acidified MRS media, we aimed to select for *Bifidobacterium* spp. that possess the ability to metabolize the HMO substrate. Previous studies have indicated the suitability of an ethanol treatment prior to plating on rich media for the effective isolation of spore-forming bacteria [63] and was thus utilised in this study. On the other hand, G.N. Anaerobic supplement offers selective isolation of Gram-negative anaerobes due to the presence of two antibiotics, vancomycin and nalidixic acid. The usage of L-cysteine improves the maintenance of the anaerobic state in the media environment. Finally, mucin was used an additive as it has been known to act as a key source of carbon to important gut microbes [64–69], including *Akkermansia muciniphila *[70, 71], *Bacteroides thetaiotaomicron *[66, 72], *Bifidobacterium bifidum *[73–75] and *Ruminococcus gnavus *[76, 77].

Minimal medium broth was prepared based on a recipe [78] from Martens EC by autoclaving (121°C for 15 mins) 100 mM potassium dihydrogen phosphate (pH 7.2) (Millipore, Merck, 529568), 15 mM sodium chloride (ThermoFisher Scientific, S/3120/65), and 8.5 mM ammonium sulphate (Sigma-Aldrich, Merck, A4915) together and then adding filter sterilised 4 mM L-cysteine (MP Biomedicals, 101685), 1.9 µM hematin (dissolved in 1 mL 1 M NaOH) (Sigma-Aldrich, Merck, H3281), 200 µM L-histidine (ThermoFisher Scientific, A10413.14), 100 µM magnesium chloride (Acros™, ThermoFisher Scientific, 447700010), 1.4 µM ferrous sulphate heptahydrate (dissolved in 10 mM HCl) (Alfa Aesar™, ThermoFisher Scientific, 014498.30), 50 µM calcium chloride (Acros™, ThermoFisher Scientific, 349615000), 1 µg/ml vitamin K3 (dissolved in ethanol) (Dr. Ehrenstorfer™, Fisher Scientific, 17988725), and 5 ng/ml vitamin B12 (Sigma-Aldrich, Merck, V6629). Filter sterilised HMO (Layer Origin Nutrition, SuperHMO^Ⓡ^ Prebiotic Mix) was added as a carbon source at 2% (w/v).

MRS pH 5.4 broth was prepared as 1% (w/v) proteose peptone (Oxoid™, ThermoFisher Scientific, LP0085B), 1% w/v beef extract (Neogen, NCM0208A), 0.5% yeast extract (BioReagents™, Fisher Scientific, BP1422500), 0.2% ammonium citrate tribasic (Alfa Aesar™, ThermoFisher Scientific, A16973.22), 0.5% sodium acetate (Alfa Aesar™, ThermoFisher Scientific, A13184.30), 0.01% magnesium sulphate heptahydrate (Acros™, ThermoFisher Scientific, 213115000), 0.005% manganese sulphate tetrahydrate (Alfa Aesar™, ThermoFisher Scientific, B22081.36), 0.2% dipotassium phosphate (Acros™, ThermoFisher Scientific, 424195000) and 1% (v/v) polysorbate 80 (Alfa Aesar™, ThermoFisher Scientific, L13315.AE). The pH was set to 5.4 using HCl before autoclaving (121°C for 15 mins). After autoclaving 0.5% filter sterilised lactose (ThermoFisher Scientific, 125090020), 0.6% of filter sterilised cysteine-HCl (ThermoFisher Scientific, A0444725) were added.

mMRS broth was prepared as 1% (w/v) proteose peptone (Oxoid™, ThermoFisher Scientific, LP0085B), 0.5% yeast extract (BioReagents™, Fisher Scientific, BP1422500), 0.2% ammonium citrate tribasic (Alfa Aesar™, ThermoFisher Scientific, A16973.22), 0.5% sodium acetate (Alfa Aesar™, ThermoFisher Scientific, A13184.30), 0.0575% Magnesium sulphate heptahydrate (Acros™, ThermoFisher Scientific, 213115000), 0.012% manganese sulphate tetrahydrate (Alfa Aesar™, ThermoFisher Scientific, B22081.36), 0.3% dipotassium phosphate (Acros™, ThermoFisher Scientific, 424195000), 0.3% potassium dihydrogen phosphate (Millipore, Merck, 529568), 0.02% sodium pyruvate (Alfa Aesar™, ThermoFisher Scientific, J61840.18), 0.0034% ferrous sulphate heptahydrate (Alfa Aesar™, ThermoFisher Scientific, 014498.30), and 1% (v/v) polysorbate 80 (Alfa Aesar™, ThermoFisher Scientific, L13315.AE). The pH was set to 5.4 using HCl before autoclaving (121°C for 15 mins). After autoclaving 0.5% filter sterilised lactose (ThermoFisher Scientific, 125090020), 0.6% of filter sterilised cysteine-HCl (ThermoFisher Scientific, A0444725), 1% ferulic acid (MP Biomedicals, 101685), mupirocin (Millipore, Merck, 69732) and hemin (ThermoFisher Scientific, 345960010), and 5% nystatin (Gibco™, Fisher Scientific, 15340029) suspension were added.

The base media BBE (HiMedia, M805), BHI (Oxoid™, ThermoFisher Scientific, CM1135), GAM (HiMedia, M1801), Rogosa (Millipore, Merck, 105413), TOS (Millipore, Merck, 43314), WCA (Millipore, Merck, W1761) and VAB (Thomas Scientific, C977D11) were prepared according to the manufacturer’s instructions. G.N. Anaerobic Supplement (HiMedia, FD002) was added at 2% concentration to specified formulations. Powdered mucin (Sigma-Aldrich, M1778) was added at 1% (w/v) to specified formulations and the insoluble fraction was not removed. The antibiotics erythromycin (Sigma-Aldrich, Merck, E5389) and kanamycin (Fisher Scientific, 10031553) were used at a final concentration of 50 µg/ml, ciprofloxacin (Sigma-Aldrich, Merck, 17850) at a final concentration of 1 µg/ml, metronidazole (Sigma-Aldrich, Merck, M3761) at 25 µg/ml, while lithium mupirocin supplement (Millipore, Merck, 69732) was added at 0.005 mg/ml final concentration, and Vancomycin hydrochloride (Sigma, SBR00001) was added at a final concentration of 0.5 µg/ml, in the relevant formulations. All media were kept inside an anaerobic cabinet (Don Whitley Scientific, Whitley A85 anaerobic workstation) for 24 hours before use to drive out any oxygen from them.

For casting plates doubled concentrations of broth were mixed with 3% agar.

### DNA extraction and PCR

The 16S rRNA gene sequence was amplified directly from the liquid grown colony by PCR with Phusion™ High-Fidelity PCR Master Mix with HF buffer (2X) (ThermoFisher Scientific, catalogue no. F531L) and 27F (5’-GAG AGT TTG ATY CTG GCT CAG-3’) and 1522R (5’-AAG GAG GTG ATC CAR CCG CA-3’) primers at 10 µM. The following program was run on a Bio-Rad CFX96 Touch System C1000 Touch Thermal Cycler: 94°C for 3 mins for initial denaturation, followed by 32 cycles of 94°C for 30 s for denaturation, 55°C for 1 min for annealing, and 72°C for 1 min for extension, and finally 72°C for 3 mins for final extension [79]. The PCR products were processed by Eurofins genomics using Sanger sequencing.

### Analysis of sequencing data

The 16S rRNA gene sequences were assigned taxonomy using BLASTn v. 2.17.0 + with the 16S rRNA gene sequence reference database [80]. This was followed by multiple sequence alignment of the 16S rRNA gene sequences using MUSCLE [81] and evolutionary analysis by Maximum Likelihood method in IQ-TREE v. 2.4.0 [82] using ModelFinder [83]. The pairwise distance matrix from the Tree generation process was used as input for hierarchical clustering in R v. 4.5.0 [84] using R-Studio v. 2025.05.1 build 513 utilizing the base R function hclust. The designation of clades was achieved using cutree function with the similarity threshold set at 99.90%.

### Generalized linear model analysis

A generalized linear model (function glm with Poisson distribution) was run in R v. 4.5.0 [84] using R-Studio v. 2025.05.1 build 513 to differentiate the effects of base medium, growth condition, ethanol treatment and added supplement on the growth and diversity of the bacterial isolates. The isolates were grouped into family-level groups *Bifidobacteriaceae*,* Bacteroidaceae*, *Enterobacteriaceae*, *Enterococcaceae*, *Clostridiaceae*, *Lachnospiraceae*,* Oscillospiraceae* and *Lactobacillaceae.* We modelled the number of colonies, and the number of species obtained per taxonomic group as a function of the medium, the supplement, the treatment, the culture condition, and the total number of colonies picked per taxonomic group as the offset, using Poisson regression. Non-significant variables were removed from the model. Estimates having p-value < 0.05 were considered significant. Estimates of effect sizes are shown as incidence rate ratios (IRRs) calculated as log of the regression estimate (β). The obtained results were visualized as a heatmap using ComplexHeatmap package in R [85].

## Results

### Phylogenetic coverage and specificity of the different media

**Fig. 1:**
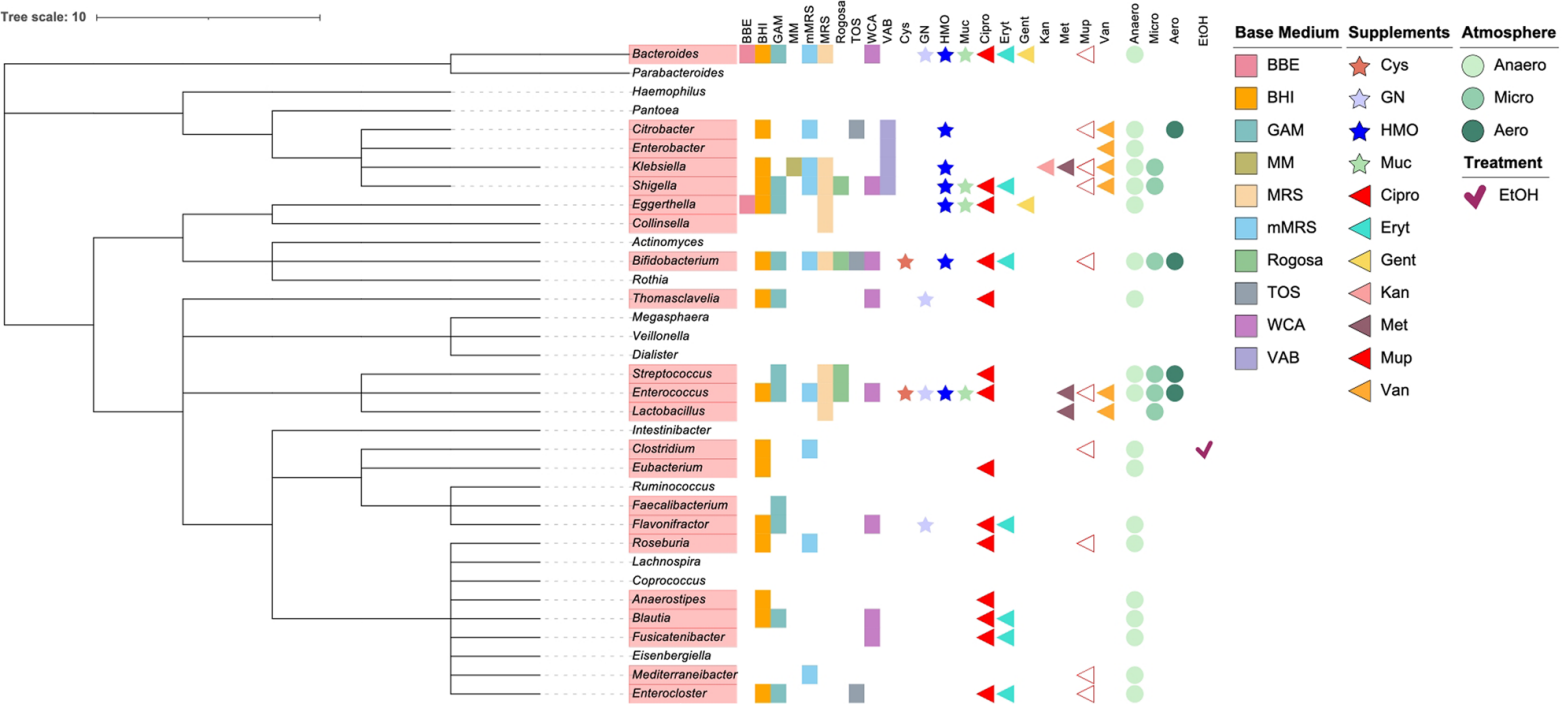
Phylogenetic tree of core bacterial genera that constitute the infant gut. These genera have been chosen based on > 50% prevalence in a cohort of 1000 Finnish infants [4]. Genera in red refers to those that have been successfully isolated in this study. The coverage of the base media, supplements, growth conditions and treatments used in this study have been shown alongside. The antibiotic supplements have been shown in triangles while the others have been depicted as stars. The atmosphere of growth has been shown as circles with ethanol treatment designated as a checkmark. Here, *BBE * Bacteroides Bile Esculin, *BHI * Brain-Heart Infusion, *GAM * Gifu Anaerobic Medium, *MM * Minimal Media, *MRS * De Man-Rogosa-Sharpe, *mMRS *modified De Man-Rogosa-Sharpe, *Rogosa * Rogosa agar original recipe, *TOS * Transgalactosylated oligosaccharides propionate, *WCA * Wilkins-Chalgren Agar, *VAB * Veillonella Agar Base, *Cipro * Ciprofloxacin, *Cys * Cysteine hydrochloride, *Eryt * Erythromycin, *Gent * Gentamycin, *GN * G.N. Anaerobic Supplement, *HMO * Human Milk Oligosaccharides, *Kan* Kanamycin, *Met * Metronidazole, *Muc * Mucin, *Mup* Lithium mupirocin, *Van* Vancomycin, *Anaero* Anaerobic, *Micro* Microaerobic, *Aero* Aerobic and *EtOH* Ethanol

The two faecal samples yielded 957 isolates that belonged to 76 different species when cultured across 40 different media formulations and growth conditions. When compared to the core bacterial genera defined as having a prevalence of greater than 50% in a cohort comprising of 1000 Finnish infants [4], this study was able to successfully isolate 63% of the core (Fig. 1). At a higher prevalence cutoff of 75%, our study was able to capture 80% of the core genera. At class level, only Negativicutes and Betaproteobacteria were not represented among the isolated colonies.

The unmodified media preparations (BHI, GAM, Rogosa, MRS and WCA) were unable to capture the entirety of the bacterial community present in the infants’ faecal samples even when considered together (31.98% of all isolated species, Table 2) in comparison to media with a supplement, of which antibiotics-supplemented media performed superior to all the other tested formulations (51.93% of all isolated species, Table 2). It was observed that antibiotic supplemented media were able to yield the most diverse communities, with mupirocin supplementation of MRS and ciprofloxacin supplementation of GAM yielding 19 and 15 different species, respectively. Among the media formulated with Human Milk Oligosaccharides (HMO), MRS additionally supplemented with Cysteine hydrochloride and Lithium mupirocin yielded 9 different isolates. When compared based on their ability to isolate bacteria belonging to the core infant gut assemblage, ciprofloxacin supplemented BHI and GAM as well as mupirocin supplemented MRS medium performed better than the others, having successfully isolated almost 23% of the 50% core consortium, each.

**Table 2:**
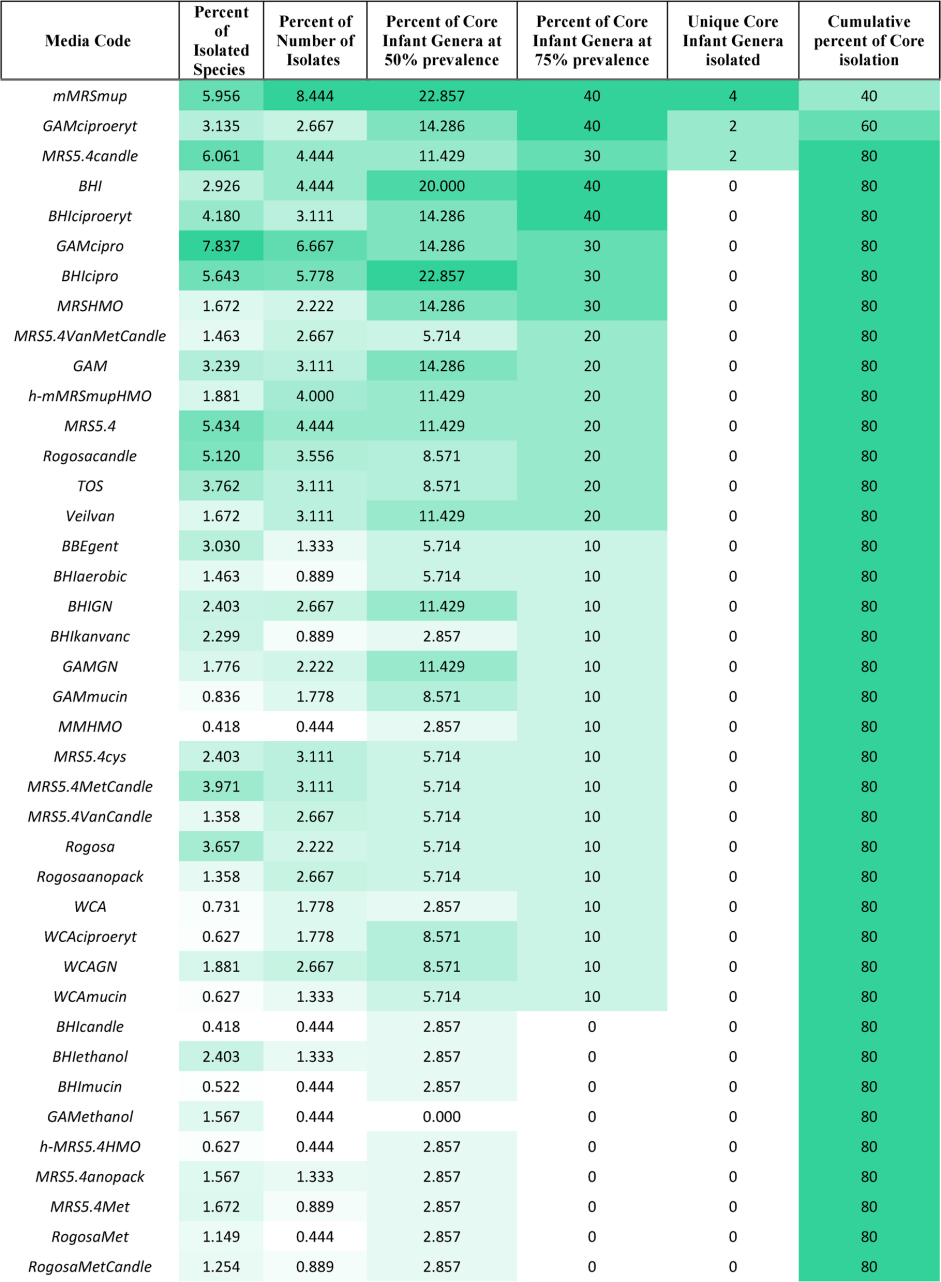
Tested media formulations and their overall efficiency, calculated as percentage of species isolated by that media formulation and fraction of total isolates obtained, in addition to percentage of core infant genera retrieved by each formulation. The colour gradient in green indicates that higher the colour intensity, higher is the efficiency of the media formulation

**Fig. 2:**
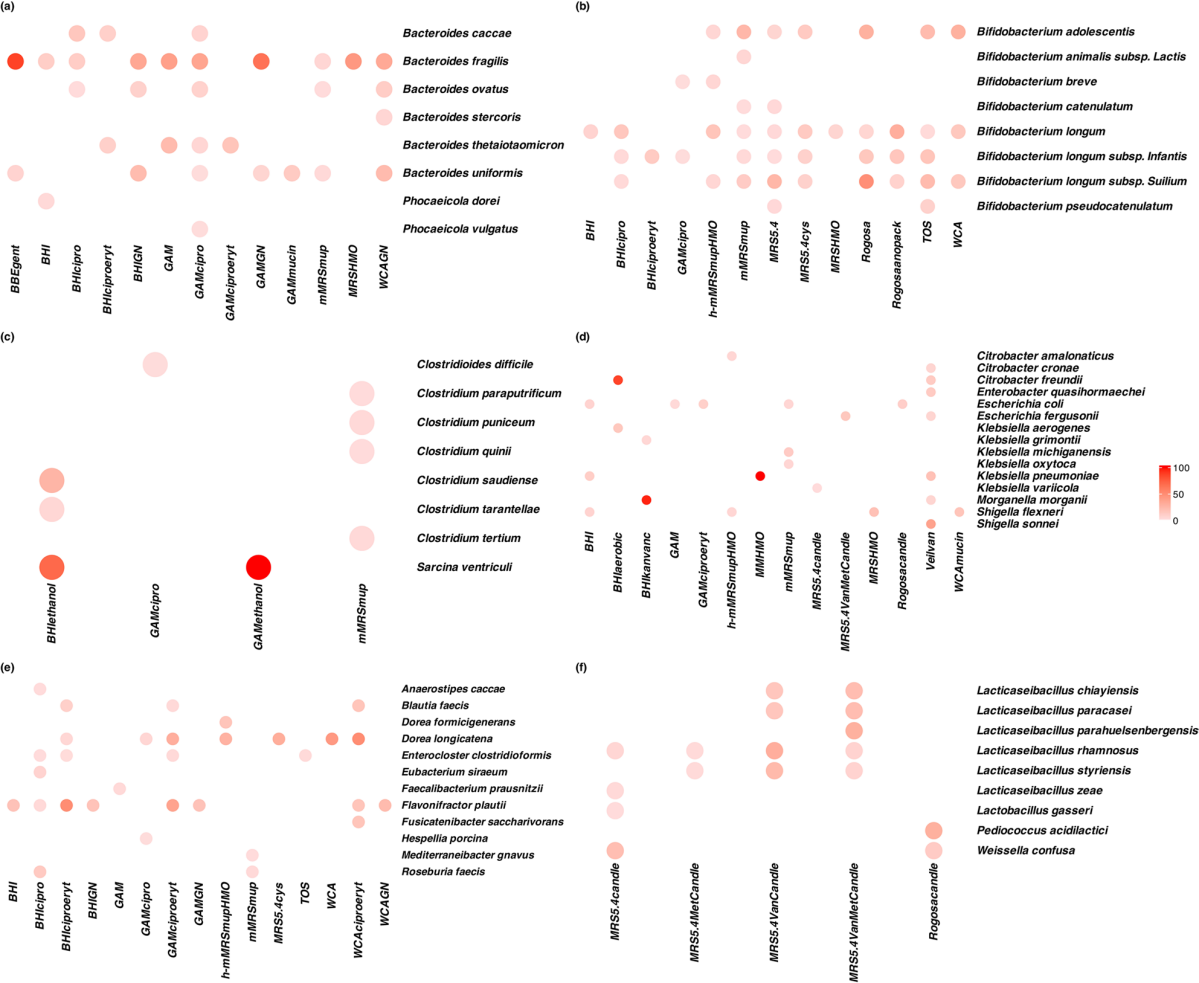
Heatmaps indicating the specificity and coverage of the media formulations used in this study. The colour gradient is based on the proportion of isolates of a bacterial species to the total number of isolates obtained on a specific medium and indicates the suitability of a media formulation towards isolating a specific bacterial species. The heatmaps show bacterial isolates belonging to (**a**) *Bacteroidaceae* (**b**) *Bifidobacteriaceae* (**c**) *Clostridiaceae* (**d**) *Enterobacteriaceae* (**e**) *Lachnospiraceae/Oscillospiraceae* and (**f**) *Lactobacillaceae* groups

Regarding species-level coverage, mMRS supplemented with mupirocin as well as MRS5.4 provided the greatest coverage for isolation of *Bifidobacteriaceae* (Fig. 2b). mMRS supplemented with mupirocin gave the best coverage for *Clostridiaceae* (Fig. 2c) while BHI supplemented with ciprofloxacin yielded the highest coverage for *Lachnospiraceae/Oscillospiraceae* isolates (Fig. 2e). For isolation of *Lactobacillaceae*, MRS medium supplemented with vancomycin and metronidazole provided the best coverage (Fig. 2f). Surprisingly, VAB with vancomycin supplementation resulted in the most diverse isolation of *Enterobacteriaceae* (Fig. 2d). GAM with ciprofloxacin returned the highest coverage for *Bacteroidaceae* (Fig. 2a). Across all bacterial groups, no single media formulation was able to isolate all the species isolated from that group (Fig. 2). On dividing the obtained isolates into intra-specific clades, some of the isolates revealed clade-wise media specificity despite majority of them exhibiting growth across multiple media (Supplementary Figure).

### Model-based prediction of media formulation specificities

**Fig. 3:**
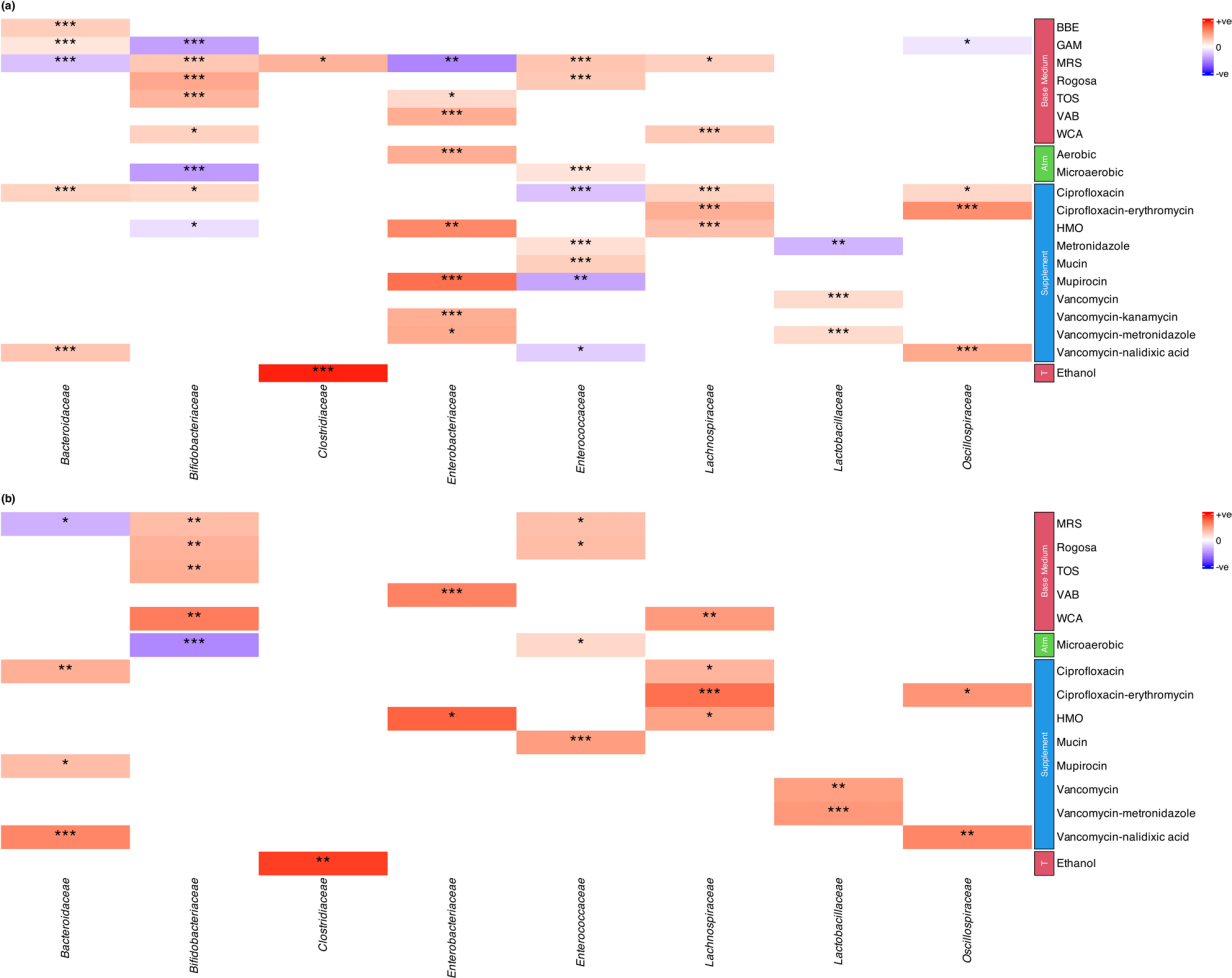
Generalized linear model predictions showing the effect of base medium, growth condition (shown as “Atm”), supplement and treatment (shown as “T”) on (**a**) selective isolation and (**b**) isolation diversity of bacterial groups. Only significant results have been shown here with estimates indicating the positive (red) or negative (blue) effect of the variable towards (**a**) growth and (**b**) diversity of the indicative bacterial group. Level of significance has been shown as ‘***’ = *p*-value < 0.001, ‘**’ = *p*-value < 0.01 and ‘*’ = *p*-value < 0.05. Exact p-values and other statistical values given in Supplementary Tables

To predict which base media, growth atmosphere, treatment and supplements would optimally support the growth of the key bacterial groups in infants, we modelled the number of colonies and number of different species per bacterial group as a function of the base medium, atmosphere, supplement and treatment (Fig. 3). All models had BHI as the reference base medium and anaerobic condition as the reference growth atmosphere.

The yield (Fig. 3a) and diversity (Fig. 3b) of *Lactobacillaceae* was predicted to increase by using vancomycin alone or in combination with metronidazole as additives to any of the base media. All *Lactobacillaceae* isolates obtained were exclusively in microaerobic conditions, but in our data set this was collinear with the addition of vancomycin-metronidazole and was therefore not statistically significant when adjusting for the antibiotic. Interestingly, metronidazole alone was predicted to have a negative effect towards *Lactobacillaceae* yield.

For obtaining *Enterococcaceae*, mucin and metronidazole supplementation in microaerobic conditions on MRS or Rogosa as base medium was predicted to increase yield and diversity. Ciprofloxacin, mupirocin and vancomycin in combination with nalidixic acid was predicted to hamper *Enterococcaceae* yield. The yield and diversity of *Clostridiaceae* was predicted to be enhanced by ethanol treatment with MRS as the base medium.

The highest yield and diversity of *Bifidobacteriaceae* could be isolated on MRS, Rogosa, TOS and WCA, with ciprofloxacin increasing yield but not diversity. Incubation in microaerobic environment was predicted to hamper both yield and diversity, while GAM and HMO were predicted to negatively impact yield only.

Ciprofloxacin and erythromycin in combination, ciprofloxacin alone, and HMO on WCA was predicted to yield the highest number and diversity of *Lachnospiraceae* isolates. The yield and diversity of *Oscillospiraceae* isolates was predicted to be best supported by either ciprofloxacin and erythromycin or vancomycin and nalidixic acid on any base medium except GAM.

*Bacteroidaceae* was predicted to grow best on BBE although this medium supported only *B. fragilis*, or GAM. No specific medium significantly increased *Bacteroidaceae* diversity. Vancomycin with nalidixic acid as well as ciprofloxacin alone were predicted to increase both yield and diversity, while mupirocin increased diversity.

The highest yields of *Enterobacteriaceae* isolates were predicted to be obtained by adding mupirocin and HMO to VAB in aerobic conditions (Fig. 3a), with HMO and VAB predicted to enhance *Enterobacteriaceae* isolate diversity. In addition, TOS was predicted as an alternate base medium that increased the yield of *Enterobacteriaceae.* Mupirocin and vancomycin in combination with either kanamycin or metronidazole was predicted to increase the *Enterobacteriaceae* yield.

## Discussion

Using a range of base media, supplements, and growth conditions, we were able to culture a large fraction of the core infant gut bacteria. The rich media did not support a broad diversity, but rather a combination of selective media preparations targeting different bacterial groups resulted in better coverage of the total community. In this study, the community of bacteria that a subset of the specialized media has been found to support has differed from their theoretical growth specificity. The results highlight the complexity of selective culturing from a diverse community. This can be attributed to the compositional variation that exists in the infant gut as compared to the adult gut microbial community, in addition to the presence of several different bacterial groups that share the ability to utilise and grow in the same nutrient and oxygen conditions.

Recent studies have highlighted the importance of BHI in microbiota research, particularly in isolating and cultivating beneficial bacteria from the infant gut [51]. However, our findings suggests that BHI may not be the optimal medium for infant gut microbiota. It did not emerge as superior in terms or yield or diversity for any of the taxonomic groups, although in combination with ethanol treatment, BHI did yield several different *Clostridium* species. For instance, it has been used in the cultivation of *Lactococcus* and various strains of *Escherichia coli*, which play significant roles in gut health and disease suppression [86] and supports our data where *Escherichia coli* isolates have been obtained from BHI media plates. Indeed, it has proven useful in the identification and characterization of endospore-forming *Clostridia* from infant faeces [87]. *Clostridium* spp. are important for understanding the infant gut microbiota, as they are more abundant than in adults [88] and are potential pathogens. Of the BHI formulations, BHI medium under microaerobic conditions (BHIcandle) and mucin-supplemented BHI medium (BHImucin) yielded the least diverse community in which only *Enterococcus faecalis* was recovered. This observation contrasts with previous studies, which support the importance of host-derived carbohydrates such as mucin in shaping the gut microbiota’s structural and functional capacities [89]. A recent study [49] found that mucin supplementation of BHI yielded stable and diverse communities from infant gut samples where they also observed that a lower amount of mucin supplementation of BHI (0.4%) yields an enhanced microbiota recovery rate as compared to a higher percentage (0.6%). The fact that we used a higher amount of mucin in this study (1%) could likely explain the observed variation from existing literature. *Enterococcus faecalis* emerged as a common generalist, able to grow abundantly on many different types of formulations.

The use of GAM in culturing gut bacteria has also been pivotal in studying the impact of the gut microbiota on infant health [90]. Previously, GAM has aided the isolation of bacteria that have been linked to reduced risks of gastrointestinal diseases and improved immune system development, providing insights into how they can be promoted through diet and other interventions [90, 91]. Here, unmodified GAM helped isolate only 7 different species while GAM after ethanol treatment (GAMethanol) yielded only isolates of *Sarcina ventriculi*. Interestingly, G.N. Anaerobic Supplemented GAM (GAMGN), which is intended to support the growth of Gram-negative anaerobes, helped obtain isolates belonging to *Flavonifractor plautii*, which is a Gram-positive anaerobe. Among the GAM-modified formulations, ciprofloxacin supplementation was quite promising as it yielded isolates from *Bacteroides*, *Bifidobacterium*, *Clostridioides*, *Dorea*, *Enterococcus*,* Streptococcus* and *Thomasclavelia*, which are all important gut bacteria of diverse phylogenetic backgrounds, indicative of broad coverage.

WCA has been shown to be effective in isolating *Bifidobacterium* spp. [92] and our results confirm WCA as one the best choices for *Bifidobacterium*. Adding supplements to the base WCA medium shifted the selection specificity of the medium towards *Bacteroides* (in WCAGN) and *Enterococcus* (in WCAmucin), although some isolates belonging to *Lachnospiraceae* and *Oscillospiraceae* groups were obtained irrespective of the additives. WCA with the G.N. supplement emerged as the medium of choice for *Bacteroides* spp.

MRS medium plays a crucial role in the study of the infant gut microbiome due to its ability to support the growth of lactic acid bacteria [91, 93]. In contrast to previous studies, we observed only 6 isolates belonging to *Lacticaseibacillus* and *Lactobacillus* out of a total of 58 isolates from base MRS medium under microaerobic conditions. The fact that the obtained microbes on MRS were dominated by *Bifidobacterium* isolates might be attributed to their compositional dominance over *Lactobacillus* in the infant gut [4]. It is interesting to note that although *Enterococcus* typically have a lower relative abundance in healthy infants, their ability to utilize similar nutritional substrates for survival, perhaps more efficiently, may explain their dominance over *Lactobacillus* in MRS media. To counteract the growth of *Enterococcus faecalis*, metronidazole supplementation has been shown to be effective [94–96] although our results deviate from this, with metronidazole supplementation yielding mostly isolates of enterococci and only 2 isolates of *Lactobacillus*. However, MRS supplementation with metronidazole and vancomycin together yielded 12 isolates of *Lactobacillus* belonging to 5 different species, followed closely by 11 isolates belonging to 4 different species on vancomycin-only supplemented MRS.

Human milk oligosaccharides are a major component of breast milk [97] and form a selective substrate for gut microbial growth, additionally having a modulatory effect on immunodevelopment of the infant and preventing adhesion of pathogens to the intestinal epithelium [98–101]. HMOs are known for their selective enhancement of *Bifidobacterium* spp. in breast-fed infants [102], but we observed only a single *Bifidobacterium* isolate on HMO-supplemented MRS media out of 16 sequenced isolates. This observation can be explained by the fact that the faecal samples utilised in this study come from non-breastfed one-year and two-year-old infants, which indicates that the HMO-utilising strains of *Bifidobacterium* have mostly been replaced by non-utiliser strains by this age of the host.

The BBE agar, formulated by Livingston, Kominos and Yee [103], is the primary isolation medium utilised exclusively for *Bacteroides fragilis *[104]. Due to the presence of oxgall and gentamycin, growth of other Gram-negative bacteria is inhibited. Indeed, we successfully obtained *Bacteroides fragilis* isolates by using BBEgent, although colonies of *Eggerthella lenta* were also found to grow. However, due to the high specificity to only *B. fragilis*, BBE does not appear as a useful medium for culturomics studies.

The major drawback of the study is the small sample size. The results are based on samples from only two infants, potentially reducing the generality of the findings due to inter-individual variation in strain composition, with potential differences in substrate requirements between strains. The fact that media preferences of strains may vary between individuals strengthens rather weakens the main conclusion that a combination of selective media is preferable over single broad media in infant gut culturomics studies. Notably, despite the small sample size, we were able to capture all major groups of infant gut bacteria, except for Negativicutes and Betaproteobacteria, and most of the core infant gut genera. Thus, the results can be used as a basis for future culturomics studies with a larger sample size.

A systematic analysis of the isolated bacteria performed in this study demonstrates that when culturing from a mixed community, the obtained isolates depend not only on the growth preferences and/or requirements of the isolate in question, but also on that of the other species present in the sample and their relative proportions. Hence, species with similar growth requirements might outcompete others on the same medium. This translates to an inability to concretely predict what species can be obtained from a media based on their theoretical growth requirements. Obtaining a very large number of isolates from the same samples using a wide range of media in this study provides a broad coverage of the infant gut microbiota and reliably assesses which bacteria are best captured by which media formulation, thus addressing the former inability. This study identifies a protocol that captures a diverse range of strains without having to pick and sequence a very large number of colonies by maximising efficiency of culturing in terms of diversity of isolates. All of these lend novelty to this study and advances existing cultivation strategies in the field of infant gut microbiome research.

## Conclusion

This study attempted to provide an answer to the choice of using a single medium with broad specificity versus targeted culturing using specialised media formulations for infant gut microbiome culturomics studies. Our results show that the diversity of isolates obtained is higher with a combination of targeted media formulations. With a combination of three targeted media formulations viz., mMRSmup, GAMciproeryt and MRS5.4candle, we were able to culture most (80%) of the core infant gut genera. This study also demonstrates that the isolates obtained often deviate from what would be expected based on the known growth requirements or data from adult faecal samples or other types of communities, such as food ingredients.

## Supplementary Information

Supplementary Material 1

## Data Availability

All 16S rRNA gene sequences have been submitted to GenBank with Accession numbers PV907206 - PV907966 which has been released to other INSDC databases, the European Nucleotide Archive (ENA) and the DNA Data Bank of Japan (DDBJ) upon acceptance.
