# Supplementary material for: Infant gut microbiota coverage by different culture media
Source: BMC Microbiol. 2026 Mar 9;26:365. doi: 10.1186/s12866-026-04854-7 (PMC13085705; doi:10.1186/s12866-026-04854-7)
Supplement: Supplementary file 1 [file 12866_2026_4854_MOESM1_ESM.odt]

Supplementary Figure. Heatmaps indicating the media specificity of the different clades of the isolates obtained in this study. The colour gradient is based on the number of isolates of a bacterial species obtained on a specific medium and indicates the suitability of a media formulation towards isolating a specific clade of the bacterial species. The heatmaps show bacterial isolates belonging to (a) Bacteroidaceae (b) Bifidobacteriaceae (c) Clostridiaceae (d) Enterobacteriaceae (e) Lachnospiraceae/Oscillospiraceae and (f) Lactobacillaceae groups.
